# Molecular Similarities and Differences between Canine Prostate Cancer and Human Prostate Cancer Variants

**DOI:** 10.3390/biomedicines11041100

**Published:** 2023-04-05

**Authors:** Demitria M. Vasilatis, Christopher A. Lucchesi, Paramita M. Ghosh

**Affiliations:** 1 Department of Urologic Surgery, School of Medicine, University of California Davis, Sacramento, CA 95718, USA; 2 Veterans Affairs (VA)—Northern California Healthcare System, Mather, CA 95655, USA; 3 Department of Biochemistry and Molecular Medicine, School of Medicine, University of California Davis, Sacramento, CA 95718, USA

**Keywords:** androgen receptor, androgen-indifferent, androgen-receptor indifferent, castration-resistant prostate cancer, dog, prostate cancer

## Abstract

Dogs are one of few species that naturally develop prostate cancer (PCa), which clinically resembles aggressive, advanced PCa in humans. Moreover, PCa-tumor samples from dogs are often androgen receptor (AR)-negative and may enrich our understanding of AR-indifferent PCa in humans, a highly lethal subset of PCa for which few treatment modalities are available This narrative review discusses the molecular similarities between dog PCa and specific human-PCa variants, underscoring the possibilities of using the dog as a novel pre-clinical animal model for human PCa, resulting in new therapies and diagnostics that may benefit both species.

## 1. Introduction

The initiation and progression of prostate cancer (PCa) in humans is initially reliant on androgen receptor (AR) signaling [1,2,3]. Directly targeting androgen ligands with androgen-inhibiting drugs (e.g., chemical castration) or decreasing their production via surgical castration has been utilized over the last 80 years in an attempt to suppress AR signaling and PCa-tumor progression [4,5,6]. Unfortunately, androgen-deprivation therapies (ADT) eventually fail for a subset of patients, and despite the presence of castrate levels of androgens, PCa progresses to an incurable form, termed castration-resistant prostate cancer (CRPC) [7,8,9]. This form of prostate cancer often continues AR signaling that is not reliant on androgen ligands (i.e., androgen independent) by way of multiple mechanisms, including mutations in the receptor or copy-number variations, and is typically treated with androgen-receptor inhibitors (ARIs) [10,11,12,13]. Eventually, new driver mutations develop in various genes in CRPC tumors, which leads to the abandonment of AR signaling altogether. Once PCa progresses, irrespective of AR signaling, it is termed androgen-receptor-indifferent PCa, a highly aggressive lethal form of the disease with poor outcomes [14,15]. Recently, the molecular characterization of CPRC and androgen-indifferent prostate cancer (AIPC) have improved our understanding of the drivers of these variants, which is critical for the identification of novel therapeutics. However, animal models for advanced forms of PCa are lacking and are wrought with limitations [16,17], which may make the approval of novel therapeutics challenging and the translation of results between species inconsistent.

The prostate of the dog, unlike that of rodents, is morphologically, histologically, and physiologically similar to that of humans and is under the control of androgens via androgen-receptor signaling [18,19,20]. Dogs also naturally develop other pathologic prostatic conditions with age, such as benign prostatic hyperplasia (BPH), in contrast to rodents, which typically develop prostatic atrophy with age [21,22]. More importantly, dogs are the among the only animals that spontaneously develop PCa, and often present with highly aggressive metastatic disease. The neutering or castration of male dogs is common in developed countries, with the majority of the male dogs in the United States undergoing this procedure [23,24]. It has been shown that castration influences PCa progression as castrated dogs develop PCa at higher rates and experience more metastases than intact dogs [25,26,27,28,29,30].

This review aims to discuss the molecular similarities between dog and specific human PCa variants to further examine the use of dogs as a suitable animal research model.

## 2. Androgen-Dependent PCa

The AR is a ligand-dependent nuclear receptor transcription factor that mediates the action of androgen ligands (e.g., testosterone) in androgen-dependent tissues in health and plays an important role in prostate organogenesis and maintenance in adulthood [31,32,33,34]. Initially, PCa is reliant on androgens and AR signaling for tumor growth and progression and is characterized by a rising prostate-specific antigen (PSA) level in humans, a marker not typically expressed in dog PCa [33,34]. This requirement for androgen-dependent growth is clinically exploited to combat advanced or recurrent PCa (following initial treatment with surgery and radiation) with the use of androgen-deprivation therapies (ADT) (i.e., androgen-ablation therapies, chemical castration) to inhibit the production or actions of androgens [35,36]. Invariably, PCa becomes resistant to ADT, and is then termed CRPC, where AR continues to signal irrespective of the presence of androgens [37,38]. Because of this continued signaling, AR targeting remains a valid treatment strategy using androgen-signaling inhibitors (ASIs) (e.g., androgen-synthesis inhibitors, androgen-receptor inhibitors). Dog PCa is most often low or null for AR expression, as well as for androgen-regulated proteins, such as NKX3.1 [39,40] (Table 1). However, opposing reports show some AR expression in intact dogs with PCa and cytoplasmic sequestration or the loss of AR in castrated dogs with PCa [41,42], making intact dogs with PCa potential models for androgen-dependent disease. As noted over 50 years ago in landmark studies performed by Huggins et al., dogs have similar pathophysiologies with respect to the androgen-dependent growth of the prostate and may still be of value to researchers [6,43].

### 2.1. Androgen-Receptor Structure

The AR in humans is a protein of 919 amino acids consisting of several functional domains, including an N-terminal domain (NTD), DNA binding domain (DBD), and a ligand binding domain (LBD) at the C-terminus [44,45]. In dogs, the AR is approximately 907 amino acids, and has homologous DBD and LBD, which are highly conserved across evolution in various species and are activated upon binding to androgen ligands [45,46].

The NTD of the AR is essential for function and the least evolutionarily conserved region of AR; however, there are still similarities between humans and dogs. The polyglutamine (i.e., polyQ, CAG) repeat region of the NTD has an average of 21–23 Qs in humans. Longer polyQ repeats are related to decreased AR transcriptional activity, while shorter polyQ repeats are related to increased AR transcriptional activity and are often associated with increased PCa risk [47,48,49]. This finding has also been recapitulated in dogs in vitro, where the introduction of AR with fewer polyQ repeats resulted in higher AR activity [50]. However, the association between shorter polyQ repeats regions and PCa is unclear in dogs: while some studies reveal that a shorter polyQ length does not predispose dogs to PCa, others show that certain breeds with shorter polyQ lengths are predisposed to developing PCa [25,51,52,53].

### 2.2. Androgen-Receptor Co-Chaperones

In the absence of the agonist ligand, the AR is bound to heat-shock proteins (HSP40, HSP70 and HSP90) and other co-chaperone proteins in a complex known as the foldosome [54,55]. Many small co-chaperone proteins with tetratricopeptide repeats (TPR), such as CYP60, PP5, FKBP51, FKB52, PP5, CHIP, and SGTA, have been shown to interact with the AR-foldosome complex [56]. The small glutamine-rich tetratricopeptide repeat-containing protein α (SGTA), is a co-chaperone of interest in PCa, and is known to stabilize the apo-AR structure in the cytoplasm prior to ligand binding. In human PCa, the SGTA, a steroid-receptor molecular co-chaperone that influences hormone action, is known to regulate AR function. The AR:SGTA ratio is increased compared to patient-matched BPH, and it is also increased when metastatic PCa tumors are compared with their primary tumors [57,58]. It is hypothesized that AR thereby overwhelms the capacity for SGTA to limit AR response to ligands and ensure the appropriate cellular localization of AR in vivo. In addition, in vitro work from this study showed that SGTA overexpression blunted the AR’s response to androgen ligands [58].

This concept has also been explored in multiple studies in dogs, which have shown that the overexpression of SGTA in vitro abates AR signaling [59,60]. Therefore, androgen-independent disease was hypothesized to be attributed to SGTA overexpression in dog-PCa-patient tumor samples by some researchers, who also subsequently showed that interference with SGTA dimerization in vitro rescues AR signaling [61].

## 3. CRPC

The CRPC is a hormone-independent but AR-dependent progression of PCa, in which ADT has failed, yet AR signaling axis is still functional and increases growth and progression. Often, this stage is characterized by more aggressive tumor behavior, including the initiation of cancer stem cell (CSC) signaling, epithelial–mesenchymal transition (EMT), and metastasis. A subset of patients develop CRPC secondary to uncontrolled AR signaling due to a mutation in the AR. Splice variants of the AR lacking the ligand-binding domain, such as ARV7, are responsible for this uncontrolled signaling in humans [4]. To date, splice variants have not been identified in dog PCa, and the use of dogs as a model for splice-variant-driven CRPC is undetermined. Moreover, TMPRSS2–ERG fusion and its alternative splice variants have also not been investigated in dog PCa, and their value as a model for this event is yet to be determined.

Additionally, mutations in AR-independent pathways are more frequent in CRPC and are thought to drive CRPC to become AR-indifferent PCa after ASI therapy. Therefore, AR presence and signaling coupled with new driver mutations in other pathways, EMT, and metastasis is are hallmarks of this progression of PCa (metastatic CRPC (mCRPC)). Because dogs often present with low or null AR expression, are castrated at an early age, develop PCa without the influence of androgens, and often present with metastasis, it has been argued that CRPC in dogs closely resembles CRPC in humans.

### 3.1. PI3K-AKT-mTOR and PTEN

The PI3K–AKT–mTOR signaling axis has been shown to play a crucial role in the development and maintenance of CRPC [62,63,64]. This pathway integrates growth signals with downstream processes that increase cell survival and proliferation [64]. The activation of PI3K by growth-factor-transmembrane signaling leads to the activation of AKT and, in turn, the autophosphorylation and activation of mTOR [65]. Subsequently, mTOR joins raptor (to form mTORC1) or rictor (to form mTORC2). The former is implicated in cellular proliferation and protein synthesis and the latter is implicated in cytoskeletal organization and cellular metabolism [66,67]. Upon activation, mTORC1 increases mRNA translation by phosphorylating eIF4E-BP1 and p70S6, with the former leading to the release of eIF4E from its hold and allowing it to join the translation-initiation complex, and the latter leading to the enhanced translation of mRNA transcripts that encode for ribosomal proteins and insulin growth factor 2 [68]. This pathway is of great interest as the overactivation of the PI3K–AKT–mTOR pathway has been implicated in cell survival in many cancer types, including PCa [69,70]. Many factors contribute to the aberrant activation of the PI3K–AKT–mTOR pathway, such as PI3K amplification, *PTEN* loss of function or deletion, and AKT, p-4EBP1, and eIF4E overexpression [69].

This entire pathway has also been observed to be upregulated in samples of dog PCa tissue when compared to the non-malignant prostatic tissue of normal intact dogs [40]. Moreover, another study found that p-mTOR and eIF4E were overexpressed in dog PCa tissues compared to normal prostate tissue, and this overexpression was correlated with a higher Gleason score in these dog-PCa-histology sections, which was similar to what has been reported in human PCa [71,72]. The deletion or loss of function pf *PTEN* has been implicated in the aberrant activation of this pathway in human PCa, and has also been shown to be lost in dog PCa [73]. Because PI3K pathway inhibitors, as well as eIF4E inhibitors, are a class of emerging therapeutics in CRPC and are being used in current clinical trials [74,75,76], dogs may be useful models for testing drugs targeting this pathway.

### 3.2. Estrogen Receptors

Estrogens have been linked to CRPC progression, and increased levels of estrogens have been correlated with more aggressive PCa [77,78,79,80]. Estrogens serve as ligands for two nuclear-receptor isoforms, estrogen receptor alpha (ERα) and estrogen-receptor beta (ERβ), and ERα has been implicated in oncogenic functions and the increased proliferation of cancer cells, while ERβ has tumor-suppressor functions because its loss leads to hyperplasia of the prostate and the initiation of PCa [81,82,83,84]. Moreover, PCa has been shown to have increased expression of ERα and decreased expression of ERβ [85,86,87]. Although ERα expression is typically observed in tumor stromal cells, one study found that ERα is also expressed in small selections of patients’ tumor epithelial cells, although this had no effect on the clinical or biochemical recurrence of disease [88]. Estrogen-receptor expression in dog PCa was explored in one study, in which the ERα was strongly expressed in normal prostate epithelium and the epithelium in BPH samples, but had decreased expression in PCa-tumor epithelium and no expression in the tumor stroma [73]. This appears to be in contrast to what is found in the majority of cases of human PCa, although additional studies are needed to confirm these findings.

### 3.3. Stem-Cell Markers

Cancer stem cells (CSC), or tumor progenitor cells, have been linked to the driving of clonal evolution and tumor heterogeneity, growth, and progression via their ability to self-renew, resist apoptosis, and differentiate [89,90,91], and have been implicated in the development of CRPC [92]. During development, basal epithelial stem cells of the prostate differentiate into luminal epithelial cells under the influence of androgens and the AR [93]. These multipotent basal progenitor cells remain quiescent in the basal epithelial layer, acting only to replenish defunct or apoptotic luminal cells [92]. However, it is believed that CSCs probably arise from these basal epithelial cells in PCa, although there is evidence that suggests that luminal-cell populations have their own lineage-restricted stem cells [92]. Classic markers of pluripotency include OCT3/4, SOX2, KLF4, c-Myc, and NANOG, which have been observed in PCa [94]. In addition, PCa CSC populations of CD44+ and CD20- often express CD133 and ABCG2 in humans. These markers are often associated with more aggressive tumors, higher Gleason scores, metastasis, and chemoresistance in humans [95,96,97,98,99].

Stem-cell-marker expression has also been explored in dog PCa [100,101]. In patient-derived dog PCa cell lines, one study found the increased expression of CD44, CD133, ITGA6, and DDX5 [100]. Moreover, these findings were recapitulated and expanded on in another study using patient-derived tumor spheroids, which were found to express OCT3/4, Nestin, NANOG, and CD44 [101]. Two additional factors, SOX9 and survivin, have also been associated with CSCs in PCa [91,102]. The factor SOX9 is repressed by androgens and is often overexpressed in CRPC as a result, while survivin is an inhibitor of the apoptosis protein (IAP) family member and is overexpressed in many cancers [103,104,105,106]. Both of these factors have also been observed to have increased expression in dog PCa [107]. Altogether, dogs appear to develop PCa with the presence of CSCs, and may serve as models for the therapeutic targeting of these tumor-progenitor cells.

### 3.4. Epithelial–Mesenchymal Transition (EMT) Markers

Epithelial cells can undergo EMT, a phenomenon that is characterized by phenotypic changes in epithelial cells, which begin to appear more spindle-shaped, in the manner of mesenchymal cells. Additionally, this phenotypic change is accompanied by biochemical changes, in which epithelial markers associated with adhesion, such as E-cadherin, are downregulated and mesenchymal lineage markers, such as vimentin, become upregulated [108]. This event leads to the higher metastatic potential of cancer cells and plays a critical role in mCRPC [109,110]. The induction of EMT has been associated with multiple molecular drivers, including TGF-β and the WNT signaling pathways [110,111,112]. This signaling induces the expression of multiple transcription factors that are known as classic regulators of EMT, such as Snail, Twist1, and zinc-finger E homeobox-binding 1 and 2 (ZEB1/2) [110].

Epithelial–-mesenchymal transition has also been shown to occur in dog PCa. One study showed that dog PCa tumors with a more undifferentiated appearance and metastatic lesions had increased TGF-β nuclear positivity on immunohistochemistry (IHC) [113]. E-cadherin has been shown to be decreased in dog PCa and is associated with higher Gleason scores in dog-PCa-histology sections, and neoplastic luminal epithelial cells have been shown to have increased expression of vimentin when compared to non-neoplastic lesions [39,114,115]. Because dogs with PCa often have aggressive disease and metastatic lesions [114], EMT is an unsurprising occurrence in their PCa progression, and this may offer insights into this process in human PCa.

### 3.5. Canonical Wnt Signaling

The canonical Wnt-signaling pathway is integral to EMT and has been shown to be important in the development of CRPC and the proliferation of CSCs in the PCa microenvironment [116]. Briefly, Wnts are secreted glycoproteins that direct development, tissue homeostasis, and stem-cell proliferation. Classically, Wnts bind to the frizzled and lipoprotein receptor-related protein (Fz/LRP) co-receptor complex of the cell membrane, which leads to the release of β-catenin from a group of regulatory proteins (i.e., the destruction complex) that encourage its ubiquitination and destruction in the proteosome. Once released, β-catenin increases in the cytosol, which allows its migration to the nucleus, where it regulates target-gene expression by interacting with the T-cell-specific factor (TCF)/lymphoid enhancer-binding factor (LEF) family of transcription factors, ultimately leading to the transcription of target genes increasing cell differentiation and proliferation [117].

For decades, Wnt signaling has been implicated in human PCa progression, and it is critical for CRPC. Furthermore, Wnt signaling has been shown to increase the proliferation and differentiation of prostate cells and promote EMT, leading to more aggressive and invasive disease [118]. This pathway has also been shown to have cross-talk with AR and is activated after ADT, encouraging and maintaining CRPC development and progression [119]. One study found increased cytoplasmic β-catenin staining, but not nuclear staining, in pre-neoplastic and neoplastic lesions compared to BPH lesions in dog PCa [114]. In contrast, a more recent study showed the overexpression of nuclear β-catenin and the loss of membranous β-catenin in dog PCa, with these findings exacerbated in metastatic lesions [120]. This was not due to the hypermethylation of APC [120], as is found in human PCa, and a different mechanism is likely to be responsible for aberrant Wnt signaling in dog PCa. Coupled with E-cadherin, membranous downregulation and increased nuclear TGF-β expression were also observed in dog PCa [39,113,114,115], Additionally, Wnt signaling is likely to be a key event in the progression of PCa in this species; therefore, dog PCa may be a good model for phases of human PCa that are reliant on this aberrant pathway (i.e., CRPC and mCRPC).

## 4. Androgen-Indifferent PCa Variants (AIPC) of mCRPC

**Figure 1:**
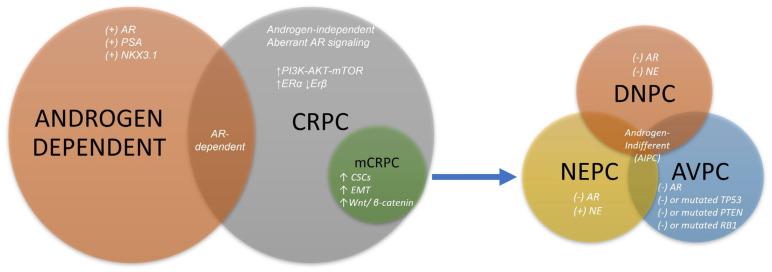
**Molecular characteristics of each PCa variant or subtype.** Prostate cancer is initially reliant on androgens binding to the AR and promoting growth. Genes commonly regulated and expressed secondary to this signaling include NKX3.1 and PSA. Castration-resistant is also reliant on aberrant AR signaling, which does not require androgen ligands. It also accumulates additional driver mutations in other genes and pathways, including PI3K-AKT-mTOR. A subtype of CRPC, mCRPC, is also known to acquire the overexpression of Wnt/B-catenin signaling, as well as increased CSCs and evidence of EMT. Lastly, AIPC is a group of PCas that are androgen-indifferent and usually AR-indifferent as well, and often arise from mCRPC. Subtypes of AIPC have specific molecular signatures. The DNPC has loss of AR- and NE-marker expression; AVPC has a loss of AR expression and mutation or loss in *TP53*, *PTEN*, or *RB1*; and NEPC usually has loss of AR expression and gain in NE markers, such as chromogranin A or synaptophysin. **Abbreviations:** AR, androgen receptor; PSA, prostate-specific antigen; ER, estrogen receptor; CRPC, castration-resistant prostate cancer; CSC, cancer stem cell; EMT, epithelial–mesenchymal transition; NE, neuroendocrine; DNPC, double-negative prostate cancer; NEPC, neuroendocrine prostate cancer; AVPC, aggressive-variant prostate cancer.

Androgen-indifferent prostate cancer (AIPC) is a form of PCa with treatment-resistant phenotypes that do not rely on AR signaling and typically arise from mCRPC following treatment with ASIs [121,122], although they may arise de novo in treatment-naïve PCa [123,124]. They are associated with an aggressive clinical course and poor treatment outcomes [14,124]. The AIPC variants of mCRPC include aggressive-variant prostate cancer (AVPC), neuroendocrine prostate cancer (NEPC), and double-negative prostate cancer (DNPC), each of which have their own distinct molecular signatures [14,125] (Figure 1). Multiple reports have shown that some dogs with PCa have molecular signatures similar to some AIPC variants. These are outlined below.

### 4.1. DNPC

The DNPC is characterized by a loss of AR and neuroendocrine (NE) markers, and is sometimes referred to as CRPC subtype DNPC (CR-DNPC) [126]. The DNPC is frequently characterized by an increase in fibroblast growth factor (FGF) and mitogen-activated protein kinase (MAPK) signaling and has upregulated genes for EMT [122]. This has been shown to occur secondary to polycomb repressor complex 1 (PRC1) activity, a histone methylase upregulating CCL2 and promoting stemness and metastasis in DNPC [127].

Similarly, dog PCa is often AR- and NE-negative with increased FGF and MAPK signaling [40], and has demonstrated the upregulation of indirect markers of EMT [39,114,115]. Therefore, dog PCa may be considered molecularly similar to human DNPC. Whether DNPC in dog PCa is reliant on PRC1 and CCL2 signaling, as it is in human PCa, is yet to be explored.

### 4.2. NEPC

Neuroendocrine prostate cancer may occur de novo or as a consequence of selection pressures on CRPC tumors from ASIs, which is termed treatment-emergent NEPC (t-NEPC) or castration-resistant NEPC (CR-NEPC) [127,128]. These tumors often lack AR and AR-regulated genes, and have an increased expression of NE markers, including pro-neural transcription factors, such as BRN2, and immunohistochemical markers, such as chromogranin A (CHGA), synaptophysin (SYP), neuron-specific enolase 2 (ENO2), and neural cell adhesion molecule 1 (NCAM1, CD56) [128,129]. In contrast, dog PCa rarely expresses NE markers [130] and NEPC has not yet been reported. This may be partly because pharmaceutical androgen-signaling therapies are not clinically used to treat dogs with PCa, thus avoiding the selection pressures that may influence CSCs to differentiate into NE cells or harbor tumor microenvironments in which NE or NE-like clones proliferate. Moreover, there are conflicting reports on whether dogs contain NE cells in the prostate at all [131,132], likely rendering the dog an unrewarding model for de novo NEPC, as well as t-NEPC.

### 4.3. AVPC

The AVPC criteria were created to identify patients predicted to have an aggressive disease that is unlikely to respond to ASI therapy and may arise de novo or after pharmaceutical treatment. Any morphological variant of PCa (e.g., small-cell prostate cancer (SCPC), adenocarcinoma, adenocarcinoma with NE differentiation) may qualify as AVPC if it has a particular molecular signature and/or meets the criteria for an aggressive clinical course [132], including signs of visceral and lytic bone metastases, lymphadenopathy, and androgen-independent progression within 6 months of starting treatment [133]. The AVPC is characterized by the deleterious loss or mutation of at least two of the three molecular tumor suppressors, *TP53*, *PTEN*, and *RB1*, and is typically responsive to platinum drugs [125]. Moreover, a study on circulating tumor cells from patients with AVPC showed that these cells have losses not only in *PTEN*, *RB1*, and *TP53*, but also in BRCA2 [134].

Dog PCa is highly aggressive and clinically similar to AVPC, but whether it has the same molecular hallmarks as human AVPC is still unclear. One study showed the decreased expression of p53 in dog PCa tissues when compared to normal and inflammatory prostatic lesions, but not complete loss [42], while another study showed no significant increase or decrease in P53 [40]. The PTEN is either downregulated or completely lost according to a few studies on dog PCa, similar to AVPC in humans [42,73]. Interestingly, *RB1* has not yet been studied in dog PCa. The loss of BRCA1 has been documented in dog PCa, but BRCA2 was not lost or downregulated according to another study utilizing RNA sequencing [40,135]. Because ASIs are not used in veterinary medicine, dog PCa may not completely match the molecular picture of human AVPC, but the molecular pictures is still incomplete, and further investigation is warranted.

## 5. Conclusions

Undeniably, there are molecular similarities between dog PCa and specific human PCa variants, although there are differences and areas that are understudied. Because the majority of dogs are neutered, present with advanced disease, and have AR null tumors, they are probably not feasible models for androgen-dependent PCa, with the exception of intact dogs in the early stages of the disease. Because of the lack of screening methods for PCa in dogs (i.e., PSA levels), establishing which intact dogs have early PCa would be challenging. Dog PCa is clinically and molecularly similar to CRPC and mCRPC, with upregulation in the PI3K-AKT pathway, Wnt signaling, EMT, CSCs, and indifference to AR, although ER expression appears to be different in this disease. Moreover, the importance of splice variants is yet to be determined in dog PCa. Lastly, regarding AIPC, it has been shown that dogs molecularly model DNPC, and may also model AVPC, but are unlikely to recapitulate NEPC. Altogether, dogs are likely to be suitable models for certain variants of advanced PCa, but not all, and additional studies are warranted to further characterize the molecular characteristics of dog PCa.

## Figures and Tables

**Table 1:** Summary of molecular characteristics of dog and human PCa.

PCa Variants	Marker of Pathway	Dog	Humans
Androgen- dependent			
	AR +	No	Yes
	NKX3.1 +	No	Yes
	PSA +	No	Yes
CRPC			
	PI3K-AKT-mTOR overexpression	Yes	Yes
	ERβ downregulation, ERα overexpression	No ^‡^	Yes
	Markers of CSCs +	Yes	Yes
	Markers of EMT +	Yes	Yes
	Wnt/β-catenin overexpression	Yes	Yes
AIPC			
*DNPC*	AR (-)	Yes	Yes
	Markers of NE (-)	Yes	Yes
*NEPC*	Markers of NE +	No	Yes
*AVPC*	*TP53* (-) or mutated	No	Yes
	*RB1* (-) or mutated	Unknown	Yes
	*PTEN* (-) or mutated	Yes	Yes

^‡^ Estrogen-receptor expression in dog PCa is present but is not identical to human PCa. Abbreviations: +, positive for expression; (-), negative for expression; AR, androgen receptor, PSA, prostate-specific antigen; CSC, cancer stem cell; ER, estrogen receptor; EMT, epithelial mesenchymal transition; AIPC, androgen-indifferent prostate cancer; DNPC, double-negative prostate cancer; NE, neuroendocrine; NEPC, neuroendocrine prostate cancer; AVPC, aggressive-variant prostate cancer.

## Data Availability

Data sharing not applicable. No new data were created in this study.
